# Biological Functions of *Dillenia pentagyna* Roxb. Against Pain, Inflammation, Fever, Diarrhea, and Thrombosis: Evidenced From *in vitro, in vivo*, and Molecular Docking Study

**DOI:** 10.3389/fnut.2022.911274

**Published:** 2022-07-12

**Authors:** Nahida Sultana, Hea-Jong Chung, Nazim Uddin Emon, Safaet Alam, Md. Tohidul Islam Taki, Sajib Rudra, Afroza Tahamina, Rashedul Alam, Firoj Ahmed, Abdullah Al Mamun

**Affiliations:** ^1^Department of Pharmacy, Faculty of Science and Engineering, International Islamic University Chittagong, Chittagong, Bangladesh; ^2^Gwangju Center, Korea Basic Science Institute, Gwangju, South Korea; ^3^Drugs and Toxins Research Divison, BCSIR Laboratories Rajshahi, Bangladesh Council of Scientific and Industrial Research, Rajshahi, Bangladesh; ^4^Department of Pharmaceutical Chemistry, Faculty of Pharmacy, University of Dhaka, Dhaka, Bangladesh; ^5^Department of Botany, Faculty of Biological Science, University of Chittagong, Chattogram, Bangladesh; ^6^Beijing Advanced Innovation Center for Food Nutrition and Human Health, China-Canada Joint Lab of Food Nutrition and Health (Beijing), Beijing Engineering and Technology Research Center of Food Additives, Beijing Technology and Business University, Beijing, China; ^7^Department of Pharmacology, Medical School, Jeonbuk National University, Jeonju, South Korea; ^8^Molecular Pharmacology Research Center, School of Pharmaceutical Sciences, Wenzhou Medical University, Wenzhou, China

**Keywords:** *Dillenia pentagyna*, pyrexia, antidiarrheal, antipyretic, thrombolytic, anti-inflammatory, molecular docking, ethnomedicinal plant

## Abstract

*Dillenia pentagyna* Roxb. is traditionally used to treat cancer, wound healing, diabetes, and diarrhea in local tribes. This study was designed to evaluate the pharmacological potentiality of this plant. *In vivo* analgesic, anti-inflammatory, and antipyretic studies of the methanol extracts of *D. pentagyna* (MEDP) leaves were performed by using acetic acid-induced nociception, formalin-induced paw licking, and yeast-induced pyrexia assay methods, respectively. *In vivo* antidiarrheal activity was carried out in mice by following castor oil-induced diarrhea and gastrointestinal transit manner. *In vitro* thrombolytic experiment was performed employing the clot lysis activity. Besides, a molecular docking study was performed by executing the software (PyRx, Discovery Studio, and UCSF Chimera). In the acetic acid-induced writhing study, MEDP possesses significant writhing inhibition in a dose-dependent manner. It showed 50.86% of maximum inhibition of pain in the case of MEDP at a dose of 400 mg/kg body weight. In the anti-inflammatory study, maximum inhibition rate was observed at a value of 59.98 and 41.29% in early and late phases, respectively, at the dose of 400 mg/kg body weight. In the case of yeast-induced hyperpyrexia, MEDP reduced hyperpyrexia in a dose-dependent manner. In the antidiarrheal assay, MEDP moderately inhibited the occurrence of diarrhea in all the experiments. In the thrombolytic study, a moderate (17.76%) clot lysis potency has been yielded by MEDP. Again, the molecular docking simulation revealed strong binding affinities with almost all the targeted proteins. The present study suggests that the MEDP possesses remarkable pharmacological activity and this finding validated the ethnobotanical significance of *D. pentagyna* as the source of pain, fever, and diarrhea management agent.

## Introduction

Pain is referred to as the response to several noxious stimuli in the immune response and is complicatedly structured through various episodes such as vasodilation, plasma extravasation, cell migration, and release of different mediators (1). In addition, bacteria and viruses trigger pyrexia which has been governed by CNS feedback mechanisms including vasodilation or sweat production and is responsible to reduce body temperature (2). Although NSAIDs are vigorously prescribed for pain, inflammation, and fever management, they can cause serious side effects following mucosal disruptions, ulcers, perforation, and blood suppression along with kidney damage, increased blood pressure, and cardiovascular complications (3). Diarrhea is one of the most prevalent diseases associated with altered bowel movement, wet stool, and abdominal pain is caused by multiple factors, namely, infections, food aversion, intestinal disorders, and also symbolic symptoms of multiple diseases like diabetes mellitus, and inflammatory diseases (4). Acute diarrhea in pediatric patients may be caused by bacteria and viruses *via* self-limiting of fluid and electrolyte replacement. Therapeutic approaches to reducing pain and diarrheal incidence are typically controlled by chemicals or medicines, but the adverse effects of such medicines present a substantial danger (4). Vascular blockage caused by a blood clot (thrombus) developed in the circulatory system due to lack of hemostasis guides to severe cascade sequels in atherothrombotic diseases including myocardial or cerebral infarction followed by death (5). About 1.5 billion (currently about 3.5 billion, i.e., 88%) of the world population are being treated by herbal medicines prepared from medicinal plants which demand new compounds discovery with promising remedial effects and negligible side effects contributing to the treatment of pain, fever, diarrhea, and thrombus (6). In structural molecular biology and computer-assisted drug design, molecular docking is an efficient process. Molecular docking is a structure-based drug design technology that simulates molecular interactions and predicts receptor-ligand binding mechanism and affinity (7). The purpose of ligand-protein docking is to predict the most likely binding modalities of a ligand to a known three-dimensional structure of a protein. Successful docking algorithms may successfully explore high-dimensional regions by using a scoring mechanism that correctly scores dockings. Docking may be used to do virtual screening on vast libraries of compounds, grade the findings, and provide structural insight into how the ligands interact with the target, all of which are immensely advantageous for lead optimization (8). Therefore, molecular docking is becoming more popular as a method for discovering new drug targets because of the growing availability of protein and nucleic acid structures. Docking structure prediction accuracy and screen hit rates have been the subject of a recent study. Docking against homology-modeled targets is now possible for a larger number of proteins as the number of experimentally known structures grows (9). Thus, research should be carried on so that these can lead us to new drug development in the future (10). *Dillenia pentagyna* Roxb. (family: Dillaneaceae) is an ethnomedicinal plant grown in the different district in Bangladesh. The plant is locally known as Banchalta, Hargaza (Bengali), Ajuli (Dhaka-Mymensingh), Argeza (Chittagong), and Ekush (Sylhet). The tribal and folk people utilize this species for the treatment of numerous diseases, for example, bone fracture (leaf), stomach cancer, pain (root), diarrhea and dysentery (leaf), pain (root), etc. (11, 12). In addition, this plant contains a number of flavonoids and triterpenes, e.g.; kaempferol, quercetin, rhamnetin-3-glucoside, isorhamnetin, and naringenin-7 galactosyl (1-4) glucoside, betulin, malic acid, lupeol, betulinaldehyde, β sitosterol, stigmasterol, and betulinic acid (13–15). Many approaches, namely, agitation, maceration, percolation, and soxhlet extraction for concentrating and extracting bioactive components from *D. pentagyna* have been demonstrated previously. Finally, based on previous reports and folkloric usages of this plant, it can be hypothesized that *D. pentagyna* can be a prospective source to develop novel therapeutics in the treatment of pain, fever, inflammation, diarrhea, and thrombosis.

In this study, phytochemical screening has been revealed to predict the elements which could enhance the pharmacological activity of methanol extracts of *D. pentagyna* (MEDP). Furthermore, we will also report the *in vivo* antinociceptive, antipyretic, antidiarrheal, and *in vitro* thrombolytic investigations of this plant extract along with *in silico* study; hence it was not accelerated previously.

## Materials and Methods

### Plant Material

In November 2019, plant leaves of the *D. pentagyna* were obtained from Mirsharai, Chittagong, Bangladesh. The plant was identified by Mr. Sajib Rudra, taxonomist, Department of Botany, University of Chittagong, Chittagong-4331, Bangladesh (Accession number: CTGUH SR 7921).

### Drying and Grinding

After the collection and identification, the leaves were washed accurately and dried under natural shade at (23 ± 2)°C for about 14 days. The dried leaves were then ground into powder using a high-capacity grinder. Powdered plant material was stored in a well-closed plastic container for further evaluation.

### Extraction of the Plant Material

According to the previously established method, from 400 g dried powder of *D. pentagyna*, 18 g crude methanol extract was obtained (16).

### Drugs and Chemicals

All drugs and chemicals used in this research were of analytical grade. To appliance the analgesic and anti-inflammatory study, acetic acid and formalin were collected from BDH Chemicals Ltd. (Poole, United Kingdom), diclofenac-Na was obtained from Incepta Pharmaceuticals (Dhaka, Bangladesh), and paracetamol and loperamide were from Square Pharmaceuticals Ltd. (Dhaka, Bangladesh). Streptokinase was obtained from Sanofi-aventis Bangladesh Ltd. (Dhaka, Bangladesh). Castor oil was supplied by Well Heath (Madrid, Spain), 10% charcoal in 5% gum acacia from Taj Scientific (Chittagong, Bangladesh), and Tween-80 and ethanol were procured from Sigma-Chemical Co. (St. Louis, MO, United States).

### Experimental Animals

To propagate experiments, Swiss albino mice (both male and female sex) aged 7–8 weeks and weighing around 25–30 g were collected from the Venom Research Centre (VRC), Chittagong, Bangladesh. Plastic cases were made to keep all animals under the 20 ± 2°C temperature and serve a 12-h light-dark cycle with the standard provision of water and food. P&D committee (Department of Pharmacy, International Islamic University Chittagong, Bangladesh) sanctioned all study protocols directed in an isolated and silent condition. The animals were acclimatized to laboratory conditions for 10 days before experimentation. The handling and taking care of animals were carried out by universal rules for the utilization and maintenance of experimental animals (17). The principles and guidelines of the Federation of European Laboratory Animal Science Associations (FELASA) were enforced to maintain the mitigation of pain and stress of the experimental models. Throughout the experiments, “3R” (Replace, Reduce, and Refine) was strictly maintained to prevent extreme pain and suffering. Experienced researchers and laboratory assistants handled the total experiment. Finally, after completion of the experiment, an anesthesia overdose [Ketamine HCl (100 mg/kg) and Xylazine (7.5 mg/kg)] through the intraperitoneal route were administered to the laboratory models followed by euthanasia (18).

### Phytochemical Screening

The phytochemical investigations of MEDP to confirm the existence of carbohydrates, alkaloids, flavonoids, tannins, terpenoids, glycosides, steroids, saponin, resin, phenol, polyphenol, protein, anthocyanin, and cholesterol were defined by the established method (19).

### Assesment of Pharmacological Actions

#### *In vivo* Bioassay

##### Preparation of Extract Solution

###### 1% Tween Solution Preparation

In total, 1 ml Tween was taken in a beaker and 99 ml distilled water was added to the beaker to prepare 1% Tween-80.

###### Dose Preparation

For preparing 200 and 400 mg/kg dose, 200 and 400 mg extract were taken, respectively, and dissolved in 10 ml 1% Tween solution.

###### Acute Toxicity Test

An acute oral toxicity study was performed according to the OECD guidelines for the testing of chemicals (20). To conduct the toxicity test, a total of five animal models were considered. They received a single oral dose of either 500, 1,000, 1,500, or 2,000 (mg/kg BW) of MEDP followed by suspension of food administration for 3–4 h and then observation of the animals was done carefully for the next 72 h. Other changes, including in skin and fur, eyes and allergic reaction, respiratory and circulatory rate, and autonomic and CNS function (excitability and sedation) were observed (21).

##### *In vivo* Analgesic Activity

###### Acetic Acid-Induced Writhing Test

The acetic acid-induced writhing test was performed by an established protocol mentioned by Emon et al. (22). Four groups of male mice (20–25 g) each having six mice were used in this experiment. MEDP (100, 200, and 400 mg/kg; p.o.) had been administered to the individual mice. After 30 min, an intraperitoneal injection of 0.7% v/v acetic acid solution was administered. In transpicuous cages, mice were positioned individually and 5 min had been allowed to endure. For 20 min, the number of acid-induced writhes had been counted. Control and standard group animals were treated with normal saline (10 ml/kg, i.p.) and diclofenac-Na (10 mg/kg, i.p.), respectively.

###### Formalin-Induced Licking Test

According to Hunskaar et al. method (23), four groups of mice, each having five mice (20–25 g) were injected 20 μl of 1% formalin prepared in 0.9% saline, subcutaneously into the dorsal hind paw and shifted instantly in a transparent box for observation. The length of response time (paw licking or biting) was once determined between 0–5 min (first phase) and 15–30 min (second phase). Animals had been administered MEDP (100, 200, and 400 mg/kg; p.o.). Diclofenac-Na (10 mg/kg, i.p.) had been given to the standard animals. Control animals had been given normal saline (0.1 ml/10 g).

##### *In vivo* Antipyretic Activity

According to Brewer’s yeast-induced fever method with some modifications (24, 25), at 0 h, the basal rectal temperature of each mouse was once recorded using a medical digital thermometer. Pyrexia was triggered with the aid of a subcutaneous injection of 15% w/v suspension of Brewer’s yeast in distilled water at a dose of 10 ml/kg body weight. After 18 h of Brewer’s yeast injection, the rise in rectal temperature used to be recorded, and only animals displaying an increase in temperature of at least 0.6°F (or 1°C) had been chosen for the study (26). The animals had been randomly divided into five groups, each group containing six mice. Group I received 1% Tween-80 in normal saline orally. Group II was given the standard drug paracetamol at the dose of 10 mg/kg orally. Groups III, IV, and V received methanol extract at an oral dose of 100, 200, and 400 (mg/kg; p.o). After the treatment, the temperature of all the mice in each group was recorded at 0, 1, 2, 3, and 4 h.

##### *In vivo* Antidiarrheal Activity

###### Castor Oil-Induced Diarrhea

The related method (27) was followed for this study with slight modification. Mice (20–25 g) fasted for 18 h. The selected mice for diarrheal tests were divided into four groups consisting of 6 mice. Group I was given normal saline (10 ml/kg) orally and designated as the control group. Group II obtained loperamide (5 mg/kg) as a standard group. Groups III–V obtained methanolic extract of MEDP (100, 200, and 400 mg/kg; p.o.), respectively. After 1 h, all groups received castor oil 1 ml each orally. Then they were positioned in cages lined with adsorbent papers and observed for 4 h for the presence of characteristic diarrheal droppings. In total, 100% was regarded as the total number of feces in the control group. The activity was expressed as % inhibition of diarrhea.


$$\%\mathrm{Inhibition}\mathrm{of}\mathrm{defecation}=\frac{\mathrm{Mo}-M}{\mathrm{Mo}}\times 100$$

Here, *M*_*o*_ = mean number of feces of the control group and *M* = mean number of feces of the test group.

###### Castor Oil-Induced Gastrointestinal Motility

This experiment was done with the strategy of a previously established method (28). All mice were divided into five groups of six mice. In total, 0.5 ml of castor oil was given orally to each mouse to produce diarrhea. After 1 h, the group I was given normal saline (10 ml/kg) orally as the control group. Group II received loperamide (5 mg/kg) as a standard group. Groups III, IV, and V received a MEDP (200 and 400 mg/kg; i.p.), respectively. After 1 h, 1 ml charcoal meal (10% charcoal suspension in 5% gum acacia) was given to all mice. The animals were then sacrificed after an hour and the small intestine was dissected from pylorus to cecum. The distance traveled by the charcoal meal from the pylorus was measured and expressed as a percentage of the total length of the small intestine from the pylorus to the cecum.


$$\mathrm{Peristalsis}\,\mathrm{index}=\frac{\mathrm{Distance}\,\mathrm{travel}\,\mathrm{by}\,\mathrm{the}\,\mathrm{charcoal}\,\mathrm{meal}}{\mathrm{The}\,\mathrm{total}\,\mathrm{length}\,\mathrm{of}\,\mathrm{the}\,\mathrm{small}\,\mathrm{intestine}}\times 100$$

$$\%\mathrm{of}\mathrm{inhibition}=\,\frac{\mathrm{Mean}\,\mathrm{length}\,\mathrm{of}\,\mathrm{small}\,\mathrm{intestine}\,\mathrm{distance}\,\mathrm{travel}\,\mathrm{by}\,\mathrm{charcoal}\,\mathrm{meal}}{\mathrm{Mean}\,\mathrm{length}\,\mathrm{of}\,\mathrm{the}\,\mathrm{small}\,\mathrm{intestine}}\,\times 100$$

#### *In vitro* Bioassay

##### *In vitro* Thrombolytic Activity

This study was conducted by following the method mentioned by Ahmed et al. (29). In total, 5 ml of venous blood had been drawn from wholesome volunteers. A total of 0.5 ml/tube of blood was dispensed in a preweighed sterile Eppendorf tube. Incubation of them at 37°C for 45 min allowed clot formation. The developed serum was eliminated except for disturbing the clot. Each tube was once again weighed to measure the clot weight. A total of 100 μl extract solutions were added separately to the tubes. As a positive control, 100 μl of streptokinase was added separately. A total of 100 μl of water was added separately to the clot of blank tubes. All the tubes were incubated at 37°C for 90 min. After 90-min incubation, the developed fluid from the clot was discarded very carefully and tubes were weighed again.

#### *In silico* Studies

##### Selection of Ligands

The compounds of *D. pentagyna* such as Kaempferol (PubChem CID: 5280863), Quercetin, (PubChem CID: 5280343), Isorhamnetin (PubChem CID: 5281654), Lupeol (PubChem CID: 259846), and Betulin (PubChem CID: 72326) were loaded in 2DSDF format, and the ligands were then minimized and turned to pdbqt format using PyRx tools to calculate the binding affinity in these targets.

##### Preparation of Targeted Proteins

The three-dimensional structures of the mu-opioid receptor-Gi protein complex (PDB: 6DDF) (30), the structure of celecoxib bound at the COX-2 active site (PDB: 3LN1) (31), human microsomal prostaglandin E synthase 1 (PDB: 3DWW) (32), kappa-opioid receptor (PDB: 6VI4) (26, 33), and tissue-type plasminogen activator (PDB: 1TPM) (34) were downloaded from the RCSB Protein Data Bank in pdb format. Gasteiger charges and hydrogen atoms were employed in a unique way to synthesize proteins. This procedure also avoided the use of any extraneous solvents. Changes in proteins were yielded in different parameters, for example, selenomethionine (MSE) was replaced by methionine (MET), Bromo UMP was replaced by UMP (U), and methylselenyl-dUMP (UMS) was replaced by UMP (U), methylselenyl-dCMP (CSL). The Dunbrack 2010 rotomer library was used to replace several of the side chains that were yet unfinished. In Chimera, the residues were retained in AMBER ff14sB mode and Gasteiger mode. As a result, all proteins have been shrunk to the least level of energy (35).

##### Docking Analysis

PyRx Autodock Vina was used to accomplish the binding interaction on the generated protein-ligand complexes (36). Docking experiments were conducted using PyRx’s semiflexible docking device. The phytochemicals were converted to PDBQT formats using PyRx AutoDock tools. The stiffness of proteins and ligands was maintained throughout this investigation. Ligand molecules had provided ten degrees of freedom (37). During the transformation, a grid box with an active site in the center was built. Finally, utilizing the BIOVIA Discovery Studio Visualizer 2020, docking sites for the best connection strategies were examined (24).

##### Statistical Analysis

Statistical analysis of data is exhibited as mean ± SEM using Graph pad prism version 5.0. Statistical significance was dictated by one-way ANOVA and was accomplished by Dunnett’s multiple comparison test. *P*-values of less than 0.05, 0.01, and 0.001 were considered statistically significant.

## Results

### Phytochemical Screening

The qualitative phytochemical screening was performed to ensure the presence or absence of secondary plant metabolites. The phytochemical screening result of MEDP showed in Table 1.

**TABLE 1:** Qualitative phytochemical screening of the methanol extracts of *Dillenia pentagyna.*

Phytochemicals	Observations
Carbohydrates	+
Alkaloids	+
Flavonoids	+
Tannins	+
Terpenoids	+
Glycosides	+
Steroids	+
Saponin	–
Resin	–
Phenol	+
Polyphenol	+
Protein	+
Anthrocyanin	–
Cholesterol	+

*(+) = Present, (–) = Absent.*

### Acetic Acid-Induced Writhing

**FIGURE 1:**
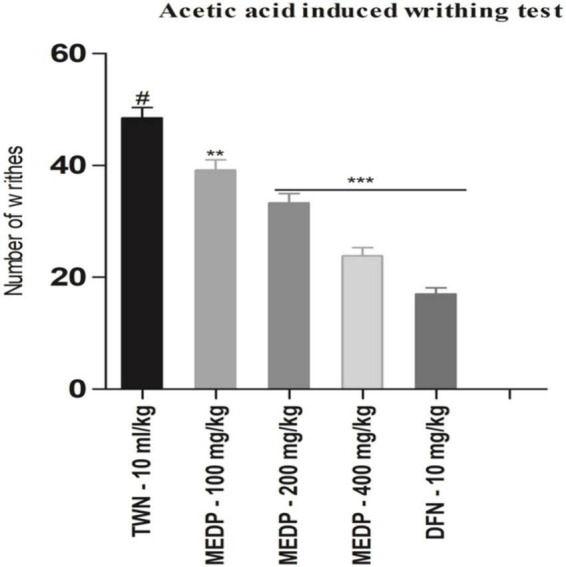
Effects of various test doses of MELM on the acetic acid-induced writhing test in mice (*n* = 6). Values are presented as mean ± SEM; one-way ANOVA followed by Dunnett’s test. *p < 0.05, **p < 0.01, and ***p < 0.001 were considered as significant compared with the control, where # is designated as control. MEDP = methanol extract of *Dillenia pentagyna*, TWN = 1% Tween-80 and DFN = diclofenac-Na.

Methanol extracts of *D. pentagyna* (MEDP) (100, 200, and 400 mg/kg) produced a dose-dependent analgesic response which moderately reduced acetic acid-induced writhing. Dose 100, 200, and 400 mg/kg produced 19.23 (^∗∗^*p* < 0.01), 31.27% (^∗∗∗^*p* < 0.001), and 50.86% (^∗∗∗^*p* < 0.001) inhibition respectively. Diclofenac-Na formed 64.94% (^∗∗∗^*p* < 0.001) of inhibition. The result of acetic acid-induced writhing has been shown in Figure 1.

### Formalin-Induced Licking

**FIGURE 2:**
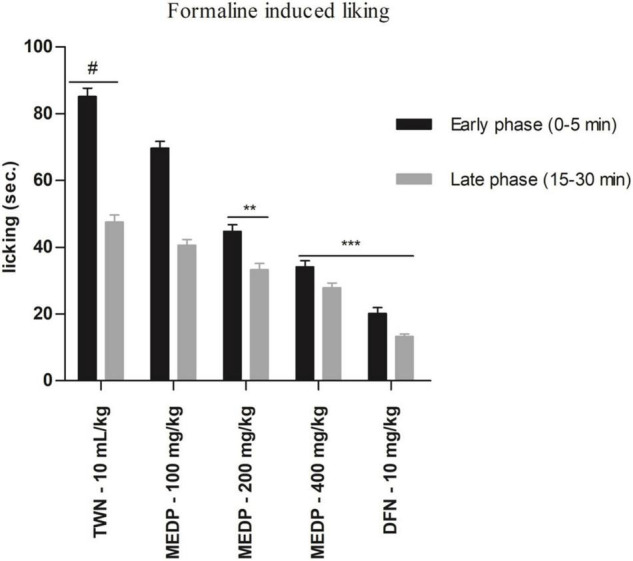
Effects of various test doses of the MELM on the formalin-induced paw licking study in mice (*n* = 6). Values are presented as mean ± SEM; one-way ANOVA followed by Dunnett’s test. **p < 0.01 and ***p < 0.001 were considered as significant compared with the control, where # is designated as control. MEDP = methanol extract of *Dillenia pentagyna* TWN = 1% Tween-80 and DFN = diclofenac-Na.

Oral administration of MEDP at different concentrations showed a moderate reduction of paw licking in both early and late phases in formalin-induced pain methods. In the early phase, 18.15%, 47.49% (^∗∗^*p* < 0.01), and 59.98% (^∗∗∗^*p* < 0.001) had been reported for MEDP 100, 200, and 400 mg/kg, respectively. On the other hand, 14.63%, 29.92% (^∗∗^*p* < 0.01), and 41.29% (^∗∗∗^*p* < 0.001) licking inhibition was recorded in the late phase for MEDP 100, 200, and 400 mg/kg, respectively. Connecting to the standard drug (diclofenac-Na) showed 76.32% (^∗∗∗^*p* < 0.001) and 72.18% (^∗∗∗^*p* < 0.001) of inhibition in both the early and late phases. The consequence of the formalin-induced paw licking test has been shown in Figure 2.

### Antipyretic Activity

Methanol extracts of *D. pentagyna* (MEDP) significantly (^∗^*p* < 0.05, ^∗∗^ *p* < 0.01, and ^∗∗∗^*p* < 0.001) reduces the fever at the dose-dependent manner. The body temperature of mice has been decreased with the administration of MEDP (200 and 400 mg/kg). MEDP 400 mg/kg showed almost similar activity to the standard drug paracetamol. The summary of hyperpyrexia reduction has been shown in Table 2.

**TABLE 2:** Antipyretic activity of the methanol extracts of *Dillenia pentagyna* in yeast induced pyrexia method.

Group	Initial rectal temperature before yeast injection (°F)	Rectal temperature at yeast injection and after the administration of sample (°F)				
		After 18 h	1st hour	2nd hour	3rd hour	4th hour
Control (Tween – 10 mg/mL)	98.90 ± 0.12	100.40 ± 0.31	100.60 ± 0.29	102.60 ± 0.20	102.30 ± 0.19	102.50 ± 0.19
Paracetamol (100 mg/kg)	98.60 ± 0.10	101.2 ± 0.31	99.20 ± 0.12**	97.90 ± 0.19***	96.90 ± 0.50***	96.30 ± 0.09***
MEDP (100 mg/kg)	98.10 ± 0.08	100.1 ± 0.50	100.0 ± 0.56	99.70 ± 0.33*	99.20 ± 0.65*	98.35 ± 0.33*
MEDP (200 mg/kg)	98.27 ± 0.06	100.4 ± 0.70	98.10 ± 0.40*	98.90 ± 0.30*	98.80 ± 0.65**	97.56 ± 0.09**
MEDP (400 mg/kg)	98.90 ± 0.07	100.8 ± 0.92	97.70 ± 0.84***	97.36 ± 0.66***	96.95 ± 0.58***	96.80 ± 0.77***

Values are expressed as mean ± SEM or percentage (n = 6). The data were analyzed by one-way ANOVA followed by Dunnett’s test. Asterisks indicated statistically significant values from control. *p < 0.05, **p < 0.01, and ***p < 0.001 compared with control. MEDP = Methanolic extract of Dillenia pentagyna.

### Castor Oil-Induced Diarrhea

In this experiment, oral administration of MEDP 100, 200, and 400 mg/kg showed significant dose-related inhibition of defecation frequency when compared to loperamide 5 mg/kg. MEDP administered at the dose of 100, 200, and 400 mg/kg showed 29.31%, 51.03% (^∗∗^*p* < 0.01), and 60.68% (^∗∗∗^*p* < 0.001) reduction, respectively, where, standard drug loperamide (5 mg/kg) yielded 78.13% (^∗∗∗^*p* < 0.001) of retardation. Result is shown in Table 3.

**TABLE 3:** Castor oil induced diarrheal test *Dillenia pentagyna.*

Treatment	Dose (mg/kg)	Average no. of feces (4 h)	% of inhibition
TWN^#^	10 mL/kg	14.5 ± 0.08	0
LPM	5	3.17 ± 0.20***	78.13
MEDP	100	10.25 ± 0.60	29.31
MEDP	200	7.1 ± 1.22**	51.03
MEDP	400	5.7 ± 0.73**	60.68

Effects of various test doses of the MEDP on the castor oil induced diarrhea test in mice (n = 6). Values are presented as mean ± SEM; One-way analysis of variance (ANOVA) followed by Dunnett’s test. **p < 0.01, and ***p < 0.001 is considered as significant compared with the control, where # is designated as control. MELM = methanol extract of Dillenia pentagyna, TWN = 1% Tween-80 and LPM = Loperamide.

### Charcoal-Induced Intestinal Transit in Mice

In this study, the results found that, after the administration of MEDP 100 mg/kg, charcoal was traveled in the small intestine up to (31.93 ± 0.83) cm. Besides, charcoal meal has been traveled almost (24.65 ± 0.73) (^∗^*p* < 0.05) cm and (18.70 ± 1.10) (^∗∗∗^*p* < 0.001) cm for MEDP 200 and 400 mg/kg, respectively. Under similar experimental conditions, only (11.23 ± 1.12) (^∗∗∗^*p* < 0.001) cm charcoal meal traveled in the small intestine of mice at a dose of loperamide 5 mg/kg. The peristaltic index and percent of inhibition in castor oil-induced gastrointestinal motility have been shown in Table 4.

**TABLE 4:** Castor oil-induced gastrointestinal motility of the methanol extracts *of Dillenia pentagyna*.

Treatment	Dose (mg/kg)	Distance travel by charcoal meal (cm)	Peristalsis index	Inhibition (%)
TWN^#^	10 mL/kg	38.60 ± 1.08	10.6	0
LPM	5	11.23 ± 1.12***	38.35	70.90
MEDP	100	31.93 ± 0.83	18.44	17.27
MEDP	200	24.65 ± 0.73*	22.27	36.13
MEDP	400	18.70 ± 1.10***	29.65	51.55

Effects of various test doses of MEDP on castor oil induced charcoal meal transit study in mice (n = 6). Values are presented as mean ± SEM; One-way analysis of variance (ANOVA) followed by Dunnett’s test. *p < 0.05 and ***p < 0.001 is considered as significant compared with the control, where # is designated as control. MEDP = methanol extract of Dillenia pentagyna, TWN = 1 % Tween-80 and LPM = Loperamide.

### Anticoagulant Activity

**FIGURE 3:**
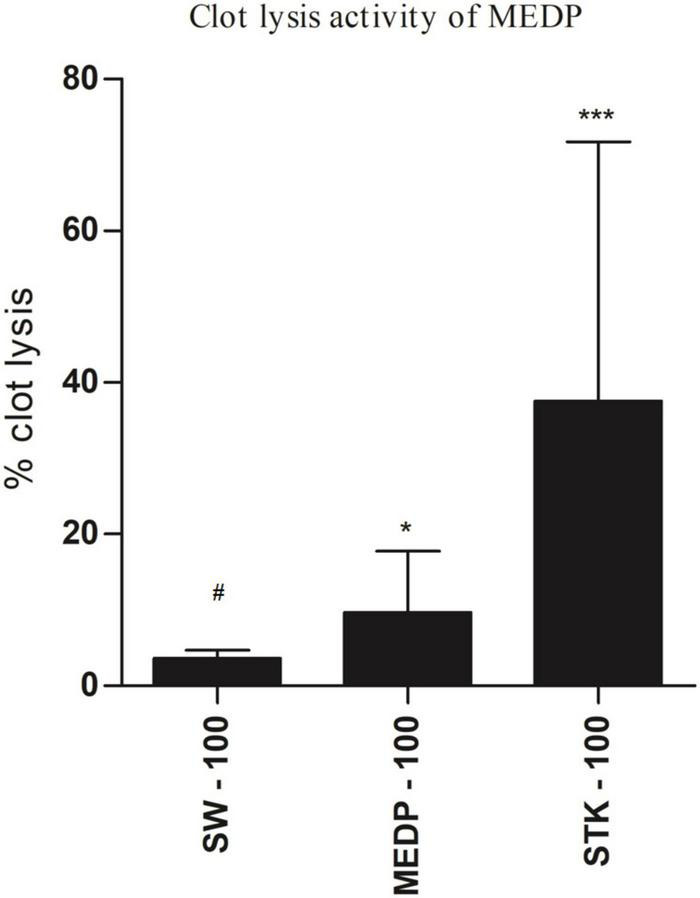
Clot lysis effects by saline water, streptokinase, and MEDP. Anticoagulant values are presented as mean ± SEM (*n* = 5); One-way ANOVA was followed by Dunnett’s test. *p < 0.05 and ***p < 0.001 were considered as significant compared with the control, where # is designated as control. MEDP = methanol extract of *Dillenia pentagyna* leaves, SW = saline water, SPK = streptokinase.

*In vitro* thrombolytic test, streptokinase (100 μl) showed 71.42 % (^∗∗∗^*p* < 0.001) clot lysis. In addition, 4.70% clots lysis was observed when the mice were treated with sterile distilled water where MEDP showed only 17.76% (^∗^*p* < 0.05) clot lysis in the clot lysis study model. A statistical representation of clot lysis percentage by negative control, positive control, and MEDP has been shown in Figure 3.

### Molecular Docking Analysis

**FIGURE 4:**
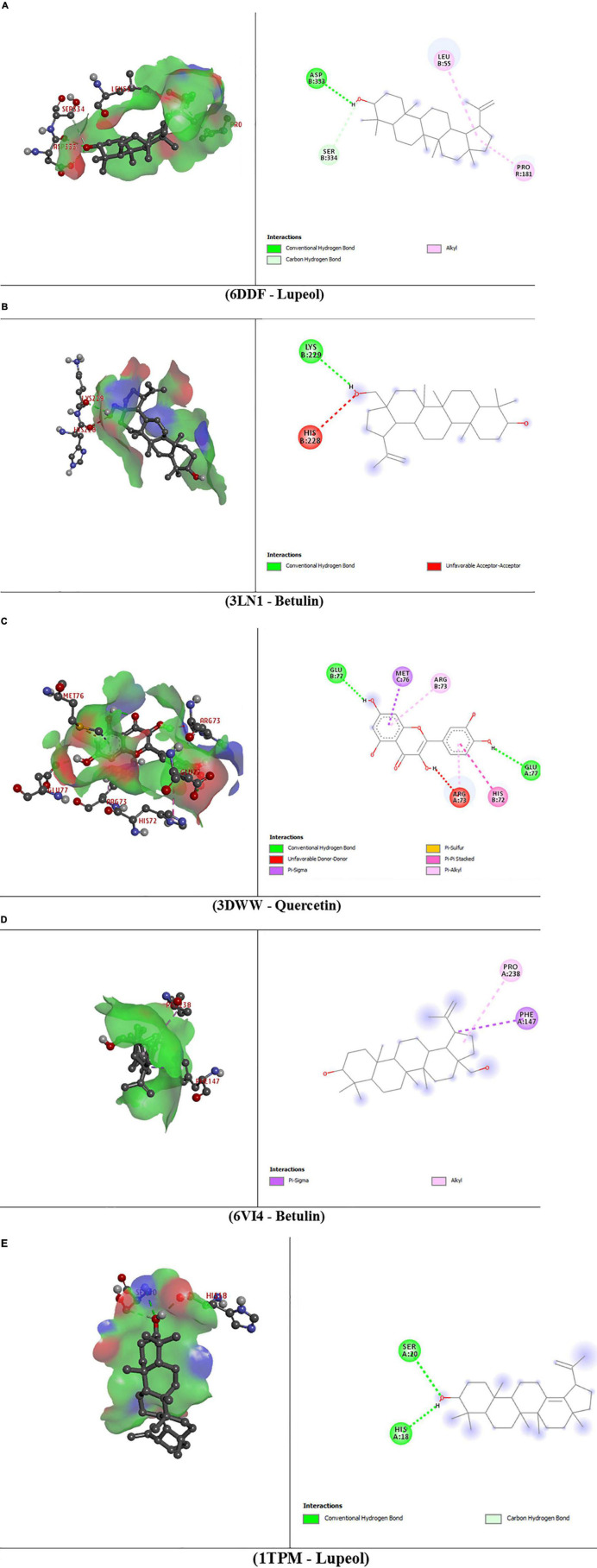
3D and 2D presentations of the best key interactions in the binding pocket for selected ligands and receptors where panel **(A)** represents the interaction of mu-opioid receptor-Gi protein complex and lupeol, panel **(B)** denotes celecoxib bound at the COX-2 active site and betulin, panel **(C)** denotes microsomal prostaglandin E synthase 1 and quercetin, panel **(D)** denotes kappa-opioid receptor and betulin, panel **(E)** denotes the interaction of tissue plasminogen activator and lupeol, respectively.

The molecular interactions of 6DDF and components of *D. pentagyna* ranked as Lupeol > Betulin > Quercetin > Isorhamnetin > Kaempferol. The docking rank for the 3LN1 receptor and selected elements is as follows: Betulin > Lupeol > Quercetin > Isorhamnetin > Kaempferol. The best hit of 3DWW with the Quercetin was possessed *via* a series of residues (glu77, met76, arg73, his72, and arg73). Besides, betulin and lupeol possessed the best binding affinity to the 6VI4 and 1TPM receptors. Betulin binds to the kappa-opioid receptor through pro238 and phe147 residues where lupeol interacted with the tissue-type plasminogen activator through his18 and ser20 residues and the binding scores of these complexes are –9.2 and 7.0 (kcal/mol), respectively. The overall interactions have been pointed out in Table 5 and Figure 4.

**TABLE 5:** The docking score of screened phytochemical’s binding at the active site of the selected proteins.

Compounds	PubChem CID	Analgesic, Anti-inflammatory and Anti-pyretic (Kcal/mol)			Antidiarrheal (Kcal/mol)	Thrombolytic (Kcal/mol)
		6DDF	3LN1	3DWW	6VI4	1TPM
Kaempferol	5280863	–7.0	–6.6	–7.0	–8.7	–5.6
Quercetin	5280343	–7.3	–6.9	–7.5	–8.8	–5.9
Isorhamnetin	5281654	–7.3	–6.7	–7.5	–8.7	–5.7
Lupeol	259846	–8.8	–7.5	–2.6	–9.1	–7.0
Betulin	72326	–8.4	–7.7	–1.4	–9.2	–5.6
Standard drugs (Ibuprofen/Indomethacin/ Loperamide/Streptokinase)	3672/3715/ 3955/9815560	–7.4	–6.4	–8.2	–7.1	–6.4

*PDB: 6DDF = Mu Opioid Receptor-Gi Protein Complex, PDB: 3LN1 = Structure of celecoxib bound at the COX-2 active site, PDB: 3DWW = human microsomal prostaglandin E synthase 1, PDB: 6VI4 = Kappa Opioid Receptor, PDB: 1TPM = tissue-type plasminogen activator.*

## Discussion

In this study, the analgesic, anti-inflammatory, antipyretic, and antidiarrheal activity of MEDP were evaluated to identify the claims which were made in traditional medicine. The results obtained from the study revealed that the MEDP produced a moderate dose-dependent inhibition of pain response. The standard drugs, however, showed a greater effect than the extract. The acetic acid-induced writhing is a manifestation of peripheral pain (38). Induction of several endogenous biochemical pain mediators like PGE2, PGI2, PGF2α (39), etc. are responsible for further stimulation of nociceptive neurons and an additional amplification of pain sensation takes place through capillary permeability (40). The pain inhibitory capability showed by the extract is a potential reflection of the apprehension of prostaglandins release mimicking the same mechanism followed by aspirin and other non-steroidal anti-inflammatory drugs (NSAIDs). A moderate exhibitory activity showed by MEDP in the formalin-induced licking test can be differentiated between the central and peripheral pain components. Pain induced by formalin is biphasic having an early phase (0–5 min) and the other late phase (15–20 min) (41). Activation of C-fiber neurons potentiates the early phase followed by the release of inflammatory mediators causing the late phase (42). A satisfactory response in both phases is reflected by centrally acting drugs. On the other hand, a peripherally acting drug is only effective in the late phase by inhibiting prostaglandin synthesis. The study result of MEDP reveals a very significant antipyretic effect in Brewer’s yeast-induced elevation of body temperature in a dose-dependent way. It is already documented that elevation of body temperature occurs by the proinflammatory cytokines productions like interleukin-1β (IL-1β) and IL-6, interferon-α (IFN-α), and tumor necrosis factor-α (TNF-α), and prostaglandins like PGE2 and PGI2 by acting on the brain (43, 44). Paracetamol and other antipyretics used in fever management work by reducing prostaglandin levels and acting on cyclooxygenase enzymes, enhancing antipyretic messages within the brain and stimulating anti-inflammatory signals at the injury site (45). A significant drop in temperature in yeast-induced pyrexia is noticed in methanol and petroleum ether fractions of *D. pentagyna* which is higher than even paracetamol and suggests that the plant possesses a significant antipyretic property. Prostaglandins produced in pyrexia are also an easy target of flavonoids (46). Several studies have confirmed the use of medicinal plants against diarrhea, i.e., antispasmodic activity slows intestinal movement, suppresses gut motility, promotes water adsorption, or decreases intraluminal fluid aggregation (47). Previously, numerous mechanisms were suggested to understand the diarrheal action of castor oil, including suppression of gastrointestinal Na^+^-K^+^ ATPase response to minimize normal fluid accumulation, amplification of adenylate cyclase, or effective secretion facilitated by mucosal cAMP (48), enhancement of prostaglandin production, platelet stimulation factor and currently, nitric oxide was believed to lead to the production of prostaglandin (49). Flavonoids and carbohydrates collected from selective conventional medicinal plants in Bangladesh have been claimed to have antidiarrheal capabilities (50). Recently, a variety of medicinal plants had been examined and it was found that the antidiarrheal action of these plants was attributed to flavonoids, alkaloids, tannins, triterpenes, saponins, reducing sugar, and sterols, existing in them (51). Again, the thrombosis or formation of the blood clot is a result of summative cascade action where damaged regions of the endothelial cell surface or blood vessel are barricaded by the platelets deposition, tissue factor, and fibrin (52) where the foundational stage is governed by platelets when the activated platelets form platelets to platelets bonds followed by further binding to the leucocytes and bringing them into a complex process of plaque formation and growth (53). So a thrombolytic agent should possess the aptness to lyse clot by disrupting the fibrinogen and fibrin and plasmin is one of the natural antithrombotic agents (54). Fibrinolysis results from the activation of cell surface-bound plasminogen to plasmin. Streptokinase, which is a bacterial plasminogen activator and a widely used thrombolytic agent, can convert additional plasminogen to plasmin (55). Phytochemicals can be prospective agents in blood clot lysis. Studies have shown that secondary phenolic compounds, such as flavonoids, have a wide range of pharmacological properties (56). Many studies are being done on flavonoids to see if they help fight free radicals, prevent heart disease and cancer, and even protect the liver from damage and inflammation (57). The immune system and inflammatory cells are significantly impacted by certain flavonoids. Flavonoids such as keampferol, quercetin, apigenin, luteolin, and hesperidin have been found to have anti-inflammatory and analgesic activities (56). Inflammation-inducing enzyme systems, such as serine-threonine protein kinases and tyrosine may be affected directly by the antioxidant capabilities of flavonoids (58). Kaempferol’s anti-inflammatory properties were attributed to a slew of mechanisms. This drug inhibits the release of IL-6, IFN-a, and TNF-a (59). The transcriptional activation of Nrf2-regulated genes and the reduction of the DNA-NF-B binding activity of the myeloid differentiation factor 88 (MDF88) are likewise reduced by Kaempferol (60, 61). Quercetin’s anti-inflammatory effects also include its capacity to prevent the synthesis of TNF-a in macrophages and the creation of IL-8 in lung cells (A549). It has been shown that quercetin reduces apoptotic neuronal cell death by inhibiting TNF-1 and IL-1 mRNA expression levels in LPS-stimulated glial cells. Quercetin also inhibits inflammation-causing enzymes [cyclo-oxygenase (COX) and lipoxygenase (LOX)] in RAW 264.7 cells and restricts LPS-induced inflammation by inhibiting Src and Syk in RAW 264.7 cells-mediated tyrosine phosphorylation of PI3K-(p85) and subsequent activation of TLR4/MyD88/PI3K (62). Lupeol’s anti-inflammatory activities were also demonstrated in studies using A23187-stimulated macrophages and triterpenes such pretreatment with lupeol to reduce PGE2 production (63). In the perspective of these observations, flavonoids and triterpenes are critical for the treatment of a wide range of health conditions, namely, chronic pain, acute inflammation, fever, diarrhea, thrombosis, and more. On the other hand, molecular docking is the process of finding the lead compound and hit compound from molecular databases using a scoring function, which has greatly increased the screening efficiency over the classic screen approach. Virtual screening has a wide range of uses. The integrated approach thrives swiftly, especially considering the exponential rise of high-throughput high-performance computing machine learning and deep learning methods (7). Considering this concept, this study also included a molecular docking study and some of the previously identified compounds of *D. pentagyna* have been docked to the mu-opioid receptor-Gi protein complex, COX-2 receptor, microsomal prostaglandin E synthase 1, and kappa-opioid receptor and the results showed prominent binding affinity of receptors and ligands. Among all compounds, Lupeol, Betulin, and Quercetin yielded the best docking scores which means these compounds have many capabilities of being the best lead compounds and better possibilities of being a good drug for the treatment of prospective diseases. With further research on cell viability tests and *in vivo* studies, this finding may have important implications in the treatment of cardiovascular diseases which is increasing at an alarming rate. Since the drugs used for cardiovascular diseases are not economical and not accessible to the greater section of society, the application of this study may be a boon for them.

## Conclusion

According to the experimental results, MEDP possessing an elevated amount of bioactive phytoconstituents can be a remarkable wellspring of analgesic and antipyretic activities with moderate anti-inflammatory, antidiarrheal, and anticoagulant therapies. The study attempts to evaluate its traditional uses in the management of pain, fever, and diarrhea though further studies are still recommended to ascertain its absolute safety and efficacy profile in the long term use and the establishment of a proper mechanism of action for exerted pharmacological activities. Future prospects of MEDP can be evaluated properly if the plant extract/respective phytochemicals can be subjected to extensive preclinical studies followed by clinical trials. Thus, for the drug-development process, future researchers can give the COX inhibitory potentials of MEDP an exclusive focus.

## Data Availability Statement

The original contributions presented in this study are included in the article/supplementary material, further inquiries can be directed to the corresponding authors.

## Ethics Statement

The animal study was reviewed and approved by the all biological activity screenings were conducted according to the ethical standards laid down in the Declaration of Helsinki 2013 (64). Animal models were handled and treated according to the principles of the Swiss Academy of Medical Sciences and Swiss Academy of Sciences and were euthanized following the Guidelines for the Euthanasia of Animals: 2013 edition.

## Author Contributions

NS, NUE, H-JC, and FA conceptualized and designed the study protocol. NS, NUE, and MTIT prepared the plant extract, designed protocols, conducted the investigations, and collected data. NUE, SA, SR, and AAM calculated the data. NUE and SA revised the manuscript. SA, NUE, NS, and SR wrote the manuscript. SA, SR, and FA supervised and monitored the research. All authors read and approved the final manuscript for publication.

## Conflict of Interest

The authors declare that the research was conducted in the absence of any commercial or financial relationships that could be construed as a potential conflict of interest.

## Publisher’s Note

All claims expressed in this article are solely those of the authors and do not necessarily represent those of their affiliated organizations, or those of the publisher, the editors and the reviewers. Any product that may be evaluated in this article, or claim that may be made by its manufacturer, is not guaranteed or endorsed by the publisher.
